# Tivozanib in renal cell carcinoma: a systematic review of the evidence and its dissemination in the scientific literature

**DOI:** 10.1186/s12885-022-09475-7

**Published:** 2022-04-09

**Authors:** Laura Caquelin, Mohamed Gewily, Wendy Mottais, Chloé Tebaldi, Bruno Laviolle, Florian Naudet, Clara Locher

**Affiliations:** Univ Rennes, CHU Rennes, Inserm, Centre d’investigation clinique de Rennes (CIC1414), service de pharmacologie clinique, Institut de recherche en santé, environnement et travail (Irset), UMR S 1085, EHESP, 35000 Rennes, France

**Keywords:** Citation analysis, Efficacy, Tivozanib, Renal cell carcinoma

## Abstract

**Background:**

Tivozanib (Fotivda) is an anti-angiogenic tyrosine kinase inhibitor that was denied access to the US market by the Food and Drug Administration (FDA). In contrast, it was granted approval by the European Medicines Agency (EMA) for the treatment of Renal Cell Carcinoma in adults. Given the conflicting decisions from these regulatory agencies, the objectives of the following study are (i) to critically review the evidence supporting the approval of tivozanib; (ii) to analyse the dissemination of this evidence in the literature by way of a citation analysis.

**Methods:**

Pivotal trials were searched by two independent reviewers using Medline, Cochrane Library, ClinicalTrials.gov and the European Public Assessment Report. The risk of bias for each trial was then inductively assessed. Articles citing any of these trials were identified using Web of Sciences. Finally, the quality of the citations was evaluated by two independent reviewers according to standard data extraction methods.

**Results:**

The search for primary evidence identified two pivotal studies: TIVO-1 upon which the FDA and the EMA decisions were based, and TIVO-3 which was conducted after the agencies’ decisions had been issued. The TIVO-1 trial presented several limitations that compromised causal inference, in relation to (i) design (absence of blinding, inappropriate comparator, and one-way crossover), (ii) poor internal consistency in the results for the primary endpoint, (iii) a discrepancy between a benefit observed for progression-free survival (HR: 0.80, 95% CI [0.64–0.99]) and the absence of difference for overall survival (HR: 1.25, 95% CI [0.95 – 1.62]). Our citation search protocol identified 229 articles that cited TIVO-1 in the 7 years following its publication, among which 151 (65.9%) citing articles discussing efficacy. Presence of spin was identified in 64 (42.4%) of these 151 citing articles, and 39 (25.8%) additional articles citing results without providing enough elements to interpret the TIVO-1 results.

**Conclusion:**

EMA’s approval was based on a single pivotal trial presenting critical limitations, rendering the results from the trial potentially inconclusive. The broad dissemination of TIVO-1 results in the scientific literature may have been affected by spin or results were presented in an inadequate critical manner.

**Supplementary Information:**

The online version contains supplementary material available at 10.1186/s12885-022-09475-7.

## Background

Tivozanib is an anti-angiogenic tyrosine kinase inhibitor that inhibits vascular endothelial growth factor receptor (VEGFR) 1, 2 and 3. Compared to the first generation of VEGFR inhibitors, tivozanib is more selective and more potent [1]. It could therefore be a more effective and better-tolerated alternative for the treatment of advanced renal cell carcinoma (RCC), an indication for which first-generation of VEGFR inhibitors, such as sunitinib, pazopanib, and sorafenib, are currently indicated [2–4]. Another reason supporting the need to find new treatments is the fact that there is no evidence of benefit on survival with any of the previously mentioned first-generation inhibitors [5].

However, the development of tivozanib was laborious. The storytelling got off to a good start with a first human study initiated in 2004 and a first pivotal study that ended only 8 years later with a statistically significant difference on its primary endpoint [6]. Subsequently, a New Drug Application was submitted in September 2012 to the Food and Drug Administration (FDA) by AVEO Oncology for the first- or second-line treatment of patients with advanced RCC. In May 2013, the FDA voted against the approval of tivozanib, arguing that the agent under investigation did not demonstrate a favourable benefit-risk ratio for the treatment of advanced RCC [7]. Furthermore, the FDA expressed serious concern about the higher death rate in the tivozanib arm compared to the control arm (sorafenib), and warned that the design of the study compromised its scientific integrity. The FDA therefore requested a second efficacy study more adequately designed to measure the overall survival (OS) effect of tivozanib. Four years after the FDA refusal, the European Medicines Agency (EMA) reached a different decision: in June 2017, the Committee for Medicinal Products for Human Use (CHMP) issued a positive opinion by majority – 25 positive votes out of 30 – for tivozanib as a first-line treatment among adult patients with advanced RCC. A divergent position, undersigned by 6 members of the CHMP, is appended to the EPAR [8].

The second pivotal study, originally demanded by the FDA, had a slow start with first inclusions in May 2016, and results were finally available in 2019 [9]. In response, the FDA recommended that AVEO Oncology should not submit a New Drug Application based on the preliminary OS data from TIVO-3 because, as the preliminary OS analysis showed a hazard ratio of 1.12, the risk of a survival detriment could not be excluded [10]. One year later, after a revised OS analysis, the FDA accepted the submission for a New Drug Application [11]. Tivozanib was finally approved by the FDA for relapsed or refractory advanced renal cell carcinoma on March 10, 2021 [12].

The exact role of tivozanib in the treatment of RCC is also debated in European guidelines. The European Association of Urology (EAU) makes a weak recommendation against offering tivozanib, noting that ‘*evidence is still considered inferior over existing 3 choices in the front-line setting*’ [13], while the European Society for Medical Oncology (ESMO) listed tivozanib as ‘*another standard of care when available*’ among good-risk patients [14].

The divergent and antagonistic opinions of these two agencies regarding tivozanib prompted us to critically review the evidence supporting its approval in this indication. In addition, as secondary sources are expected to provide a reliable summary of the level of evidence, we aimed to analyse the dissemination of this evidence in the literature using a citation analysis.

## Methods

In this review, we sought to be descriptive and exploratory. No protocol was registered on dedicated platforms prior to the searches.

### Search strategy

In April 2020 (with an update on December 14, 2020), we performed an electronic search of Medline, Google Scholar and Cochrane Library databases with the search terms ‘tivozanib’, and ‘renal cell carcinoma’ to identify pivotal studies that led to the approval of tivozanib (Supplementary table 1). Two reviewers (L.C. and C.L.) independently reviewed the titles and abstracts of all citations. Potentially relevant studies were examined in full-text. The predefined inclusion criteria were: (i) participants: patients with advanced RCC, (ii) intervention: tivozanib whatever the dose (iii) comparator: placebo or active treatment (iv) outcome: any outcome (v) study: randomized clinical trial designed to demonstrate clinical efficacy of tivozanib. Articles reporting results of pivotal studies supporting tivozanib approval are referred to as ‘*target articles’*.

We also searched ClinicalTrials.gov using the term ‘tivozanib’ and examined the European public assessment report (EPAR) for Fotivda [8]. In the absence of a marketing authorisation, no documentation from the FDA was available.

Risk of bias was inductively assessed by two reviewers for each study. The two reviewers appraised the methodology qualitatively and discussed thoroughly before agreeing on the existence of any potential limitations. Shortcomings highlighted in the CHMP members’ divergent positions were also taken into consideration.

### Citation search

Using Web of Science, we identified all articles – published before December 16, 2020 – that cited any target paper. All articles that had cited the target paper were evaluated regardless of the publication date, publication type, or publication language. Articles citing the ‘*target article’* are referred to as ‘*citing articles.’* After evaluation by two independent reviewers, citing articles that did not provide any information on TIVO-1 trial results or tivozanib efficacy were excluded from further analyses.

### Data extraction for ‘*citing articles’*

After a critical analysis of the primary evidence, one reviewer author (L.C.) drew up a standard data extraction form that was subsequently pilot-tested on 10 cited articles and validated by a second reviewer author (C.L.) prior to data extraction. Then two authors (L.C., M.G., W.M., or C.T.) independently extracted information about the studies included. Disagreements were resolved by discussion between the two reviewers or after referral to a third reviewer (C.L.) until a consensus was reached. For each ‘*citing article’*, the following information was collected: (i) the potential existence of at least one author exhibiting conflict of interests with Aveo Pharmaceuticals or Astellas (a marketing partner); (ii) the mention of clinical study characteristics (i.e. randomisation, masking, primary outcome, numbers of patients included); (iii) the mention of results concerning progression-free survival (PFS); (iv) the mention of results concerning OS; (v) and presence of ‘spin’.

Spin is defined as the use of specific reporting strategies – either intentional or unintentional – that distort the interpretation of results and misguide readers [15]. To our knowledge, lists of spin are only described for original articles – such as randomized clinical trial reports or systematic reviews and meta-analyses [16] – but not for citing articles. In our study, spin was defined in case of (i) misleading citation that over-estimates the beneficial effect of tivozanib, (ii) inadequate reporting of the limitations or bias identified during the analysis of the evidence. Spin that overestimated the efficacy of the control treatment was not considered.

### Data analyses and co-authorship network analysis

This study was intended to be primarily descriptive in nature. Descriptive analyses were performed using R software, version 4.0.3. To identify possible clusters of citing authors, the co-authorship network was visualized using the ‘network’ R package.

## Results

### Primary evidence

**Fig. 1:**
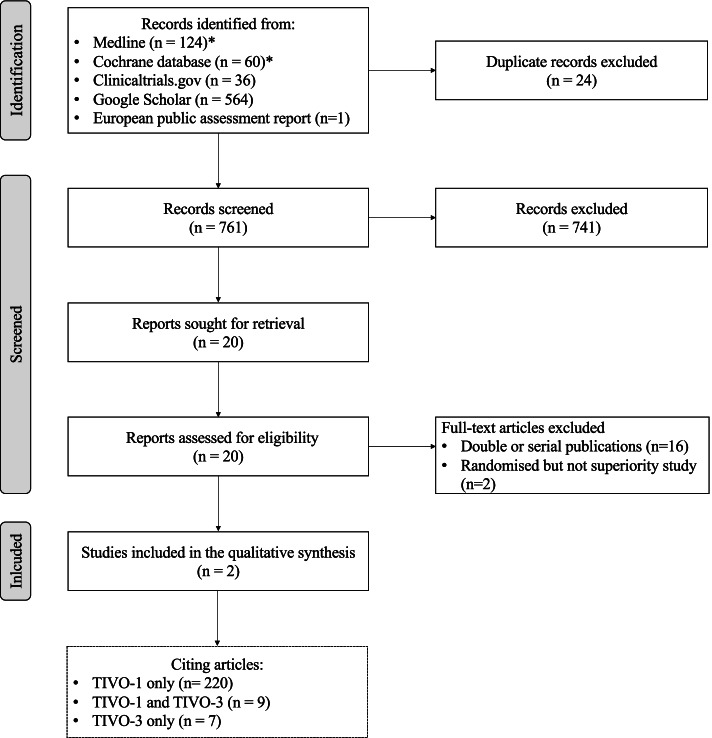
Flow chart

The searches yielded a total of 761 records (Fig. 1). Of these, 741 were excluded by review of the title or abstract because they did not meet the selection criteria. On inspection of titles and abstracts, 20 potentially eligible records were retrieved and the full texts were analysed. After examination of the full text of the remaining 20 records, a further 18 records were discarded. Two studies were included in the qualitative analysis (Table 1). The first one (TIVO-1: NCT01030783) was published in 2013 [6] and was therefore the only pivotal study available at the time of EMA approval. The second one (TIVO-3: NCT02627963), which ended 2 years after approval, was published in early 2020 [9] and is now being given consideration by the FDA.

**Table 1:** Main characteristics and results from pivotal RCTs on tivozanib for the treatment of advanced RCC

	NCT01030783	NCT02627963
	TIVO-1	TIVO-3
Design	Multicentre, randomised, controlled, open-label study + one-way crossover	Multicentre, randomised, controlled, open-label study
Availability of protocol and Statistical Analysis Plan on *ClinicalTrials.gov*	No	Yes
No. of patients	517	350
Inclusion	Feb 2010 – Aug 2010	May 2016 – Aug 2017
Prior treatment	70% no prior treatment 30% prior systemic therapy	100% ≥ 3^rd^ line therapy
Investigational drug	Tivozanib: 1.5 mg once a day (3 weeks on and 1 week off)	Tivozanib: 1.5 mg once a day (3 weeks on and 1 week off)
Control Drug	Sorafenib: 400 mg twice a day (continuously)	Sorafenib: 400 mg twice a day (continuously)
PFS^a^ (primary endpoint)		
Median tivozanib	11.9 months	5.6 months
Median sorafenib	9.1 months	3.9 months
HR [95% CI]	0.80 [0.64–0.99]	0.73 [0.56–0.94]
OS (secondary endpoint)		
Median tivozanib	28.8 months	16.4 months
Median sorafenib	29.3 months	19.7 months
HR [95% CI]	1.25 [0.95–1.62]	0.99 [0.76–1.29]
ORR^a^ (secondary endpoint)		
Tivozanib	33% [27 – 39]	18% [12–24]
Sorafenib	23% [18–29]	8% [4–13]
Reference	Motzer et al., 2013 [6]	Rini et al., 2020 [9]

^a^according to the blinded independent radiological assessment

*ORR* objective response rate, *OS* overall survival, *PFS* progression-free survival

### The TIVO-1 trial

#### Design of TIVO-1

TIVO-1 was an open-label randomized controlled trial in which 517 patients with advanced or metastatic RCC were allocated to either tivozanib (*N* = 260) or sorafenib (*N* = 257). The main eligibility criteria included (i) age ≥ 18 years; (ii) prior nephrectomy; (iii) histologically confirmed RCC with a clear-cell component and recurrence or metastases; (iv) treatment-naive or treated with prior systemic therapy. The primary endpoint was PFS assessed using the Response Assessment Criteria In Solid Tumors (RECIST) imaging criteria and reviewed by independent radiologists who were blinded to the patients’ treatment assignments. Secondary endpoints included OS, and the objective response rate (ORR) defined as complete response plus partial response. The drugs under study – tivozanib or sorafenib – were continued until progression of the disease. In case of progression, patients could access a next-line therapy; in particular, patients initially treated with sorafenib could be switched to tivozanib in an optional single-arm extension study (NCT01076010) which was later published as a separate paper [17].

#### Results of TIVO-1

The main efficacy results (OS and results reported by the blinded independent radiology review) are summarized in Table 1. The median PFS was significantly longer with tivozanib than with sorafenib (11.9 vs 9.1 months, respectively; HR: 0.80 [95% CI: 0.64–0.99]; *p* = 0.042). The ORR was comparatively higher in the tivozanib arm (33% [27 – 39] vs 23% [18–29]; *p* = 0.014). However, the results for OS were not significantly different between the two arms, and showed a trend towards longer survival in the sorafenib arm (28.8 vs 29.3 months, respectively; HR: 1.25 [0.95 – 1.62]; *p* = 0.105). Therefore, the TIVO-1 results support the efficacy of tivozanib on PFS, ORR but not on OS.

#### Imbalance in post-study treatments

Sixty-five percent of the patients in the sorafenib arm received a next-line therapy compared to only 26% of the patients in the tivozanib arm. This difference was primarily caused by the fact that patients who had disease progression in the sorafenib arm were allowed to cross over to the tivozanib arm (extension study: NCT01076010). It was also in part caused by the non-availability of targeted agents in eastern countries outside a clinical trial. Thus, this imbalance in post-study treatments probably contributed to the conflicting results between PFS and OS. However, there is nothing to rule out the hypothesis that the lower OS in the tivozanib arm could be linked to the toxic effect of this drug. Generally speaking, one-way crossover designs compromise the interpretation of OS. Several statistical approaches could be used to limit the resulting bias. But such methods have to be planned a priori in the study protocol and must not be used to ‘*ascertain that a treatment confers an OS advantage when this is not apparent in an analysis that does not (strongly) depend on unverifiable assumptions such as an “ITT-analysis”'* [18].

Additionally, the divergent position states that ‘*it is also of concern that a large proportion of patients started a new anti-cancer therapy before progression*’ [8]. But neither in the EPAR nor in the TIVO-1 article, we were unable to identify the number of patients concerned, so we could not take account of this limitation.

#### Internal consistency concerning the primary endpoint

In the divergent positions appended to the EPAR, the CHMP members were also concerned by the lack of internal consistency in the results for the primary endpoint. Indeed, the exploratory results of subgroup analyses according to the geographical region suggested that the efficacy was driven by the subgroup of patients enrolled in North America and Western Europe who made up only a minority of the patients (40 patients [7.7%]). Likewise, predefined subgroup analyses according to the ECOG performance status suggested that the efficacy was driven by the subgroup of patients with ECOG performance status 0, who accounted for half of the patients (255 patients [49.3%]). Unfortunately, interpretation of these subgroup analyses is limited by the absence of interaction tests.

#### Choice of comparator

Sorafenib is approved for the treatment of patients with advanced RCC who have not responded to prior interferon-alpha or interleukin-2 based therapy, or are considered unsuitable for this therapy [2]. Treatment with sorafenib has never been indicated for the treatment of advanced RCC in first-line, neither in 2009 [19] nor in the most recent guidelines [14]. However, 70% of the patients included in the study were treatment-naïve. Thus, the choice of the comparator is questionable when used to assess the efficacy of tivozanib in the first-line treatment of adult patients with advanced RCC.

#### Differences in dose reductions

In case of adverse events, the tivozanib dose was reduced from 1.5 mg once a day to 1.0 mg once a day (66% of the starting dose, with no option for further reduction) and the sorafenib dose was initially reduced from 400 mg twice a day to 400 mg once a day and then to 400 mg every other day (50% and 25% of the starting dose, respectively). In addition, the proportion of patients with dose reductions due to adverse events was greater in the sorafenib group (43%) than in the tivozanib group (14%), and this could result from the different rules for dose reduction in case of hypertension. Therefore, more patients in the sorafenib arm decreased their starting dose, and had a larger dose reduction than in the tivozanib arm. Unequal dose reductions could induce a systematic bias in favour of tivozanib.

### The TIVO-3 trial

#### TIVO-3 design

TIVO-3 was an open-label randomized controlled trial in which 350 patients with advanced or metastatic RCC were allocated to tivozanib (*N* = 175) or sorafenib (*N* = 175). The eligibility criteria were similar to those for TIVO-1, except for previous therapy: all patients had previous unsuccessful treatment with two or three systemic regimens (one of which included a VEGFR tyrosine kinase inhibitor other than tivozanib or sorafenib).

#### TIVO-3 results

The main efficacy results are summarized in Table 1. On the basis of the blinded independent radiology review, the median PFS was significantly longer with tivozanib than with sorafenib, while OS did not significantly differ between treatment groups.

#### TIVO-3 limitations

Compared to TIVO-1, the same limitations concerning unequal dose reductions (34% versus 50%) and choice of comparator are found for TIVO-3. Indeed, evidence that sorafenib is active as a second-line treatment has been assessed following cytokines [2]; but to the best of our knowledge, studies that assessed the efficacy of drugs among patients who had received previous VEGFR-targeted therapy, used sorafenib as the ‘standard’. Therefore, it is not clear whether sorafenib is a robust comparator for later lines of therapy.

Unlike TIVO-1, the percentages of patients who received subsequent anticancer therapy are more balanced: 40% in the tivozanib group versus 47% in the sorafenib. Therefore, the OS analysis was not compromised by an imbalance in post-study treatments. Even so, no OS benefit was identified and due to the large confidence interval, a detrimental effect on OS could not be completely ruled out.

### Citation searches

Completed after the EMA approval, TIVO-3 was not included in the citation analysis. Therefore, Motzer et al*.*, 2013 [6] – corresponding to TIVO-1 – was the only cited article included in the citation analysis. As mentioned above, because of several study limitations, the results of this trial are difficult to interpret and inconclusive.

On December 16, 2020, the citation search identified 229 articles citing the target article among which 5 were not included because full-texts were not available. Seventy-three (32.6%) were excluded from further analysis because they did not discuss tivozanib efficacy. The main characteristics of the 151 remaining articles are presented in Supplementary table 2.

#### TIVO-1 study description

Among the 151 citing articles discussing the efficacy of TIVO-1, the number of subjects was mentioned in 54 (35.8%) citing articles, randomization in 52 (34.4%), one-way crossover in 43 (28.5%), and open-label in 16 (10.6%). PFS was clearly referred to as the primary outcome in only 27 (17.9%) citing articles.

#### Dissemination of the evidence on PFS and OS

**Fig. 2:**
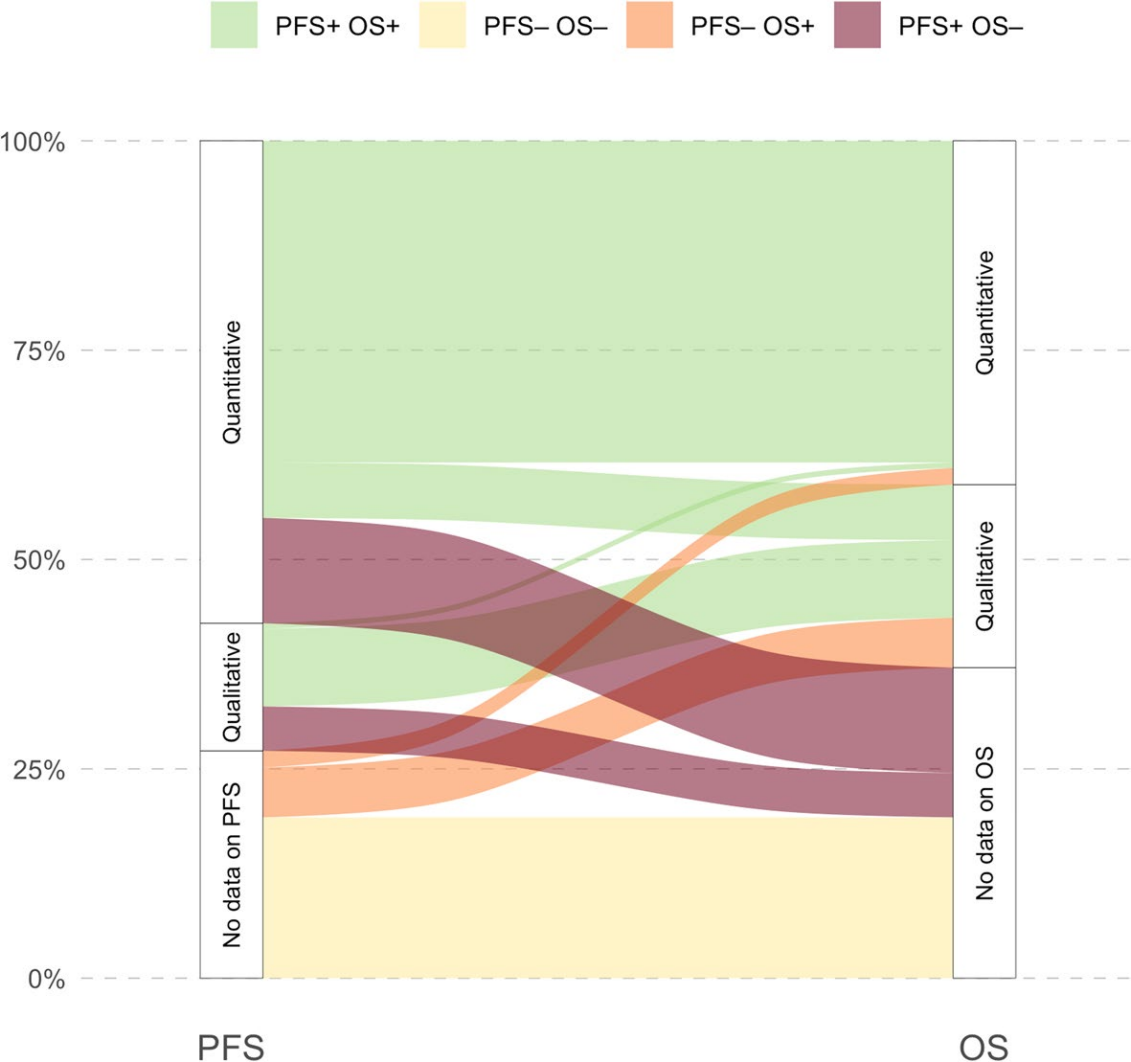
Alluvial diagram showing mentions of the PFS and OS results of TIVO-1 in citing articles. Green: 55.0% publications reported results for both PFS and OS; yellow: 19.2% publications reported neither PFS nor OS; orange: 7.9% publications reported only OS; red: 17.9% reported only PFS. When they are reported, PFS and OS are described in quantitative manner (HR, median or both) in 79.1% [87/110] and 65.3% [62/95]

Results on PFS or OS were presented in 110 articles (72.8%) and 95 (62.9%), respectively (Table 2). PFS results were expressed qualitatively and quantitatively in 87 articles (57.6%). Results on OS were expressed qualitatively in 79 (52.3%) and quantitatively in 62 (41.1%) (Fig. 2). Among the 79 articles that qualitatively commented on OS results, 44 (55.7%) articles described the OS analysis using the phrase ‘trend towards longer survival’ to describe the differences between the sorafenib arm and the tivozanib arm, and 42 (53.2%) articles referred to ‘no difference’ between the sorafenib and tivozanib arms.

**Table 2:** Dissemination of PFS and OS results across the 151 citing articles

	PFS	OS
Either qualitative or quantitative results	110 (72.8%)	95 (62.9%)
Qualitative	87 (57.6%)	79 (52.3%)
Quantitative	87 (57.6%)	62 (41.1%)
Median	80 (53.0%)	57 (37.7%)
HR	57 (37.7%)	38 (25.2%)
*p*-value	63 (41.7%)	39 (25.8%)

#### Spin and uncritical citations

Spin was identified in 64 (42.4%) citing articles that inappropriately reported the results of TIVO-1. More specifically, (i) in 39 articles (25.8%), tivozanib was presented as a treatment of undisputed efficacy for RCC, (ii) in 26 articles (17.2%), OS results were omitted while PFS results were described, and (iii) in 10 articles (6.6%), OS results were attributed solely to the crossover design. Supplementary table 3 gives examples of wordings extracted from the citing articles. Among the 87 articles without a spin citation, 39 (44.8%) articles cite PFS and OS results in their raw form, i.e. without contextualisation or analysis.

#### Marketing authorization

While all citing articles were published after the FDA refusal, this information is mentioned in only 38/151 publications (25.2%). Among the 72 articles published after the EMA approval (i.e. August 2017), 33 (45.8%) provide this information.

### Co-authorship network

The 151 articles citing the results of the target article (*Motzer *et al*.*, [6]) involve 722 authors with a median number of articles of 1 (range: 1 – 10). Interactions between authors who cite *Motzer *et al. [6] are presented in the co-authorship network in supplementary Fig. 1. A cluster of almost a third of the authors was interconnected for having published in collaboration 42.4% of the citations. The other half of the citations were published by groups of independent authors.

## Discussion

Our systematic review of available evidence highlighted two randomized pivotal trials evaluating tivozanib for the management of RCC, one using the drug as a first- or second-line therapy [6] and the other as a third- or fourth-line therapy [9]. The results of TIVO-1 were released in 2013, but critical limitations rendered this trial inconclusive. The limitations were: inappropriate comparator, one-way crossover, absence of blinding, and a lack of internal consistency in the results for the primary endpoint. Although the difference observed for OS was not statistically significant, the trend towards longer survival in the sorafenib arm could be interpreted in many ways. Firstly, it could be due to random variations, as the confidence interval was wide. Secondly, it could be attributed to the imbalance across post-study treatments, partly due to the one-way crossover design. While one-way crossover design is widespread in oncology trials, its use is a controversial issue, mainly because it could confound estimates of overall survival [20, 21]. EMA itself considers that ‘*cross-over should generally be avoided in order to meet the objectives of the trial. If nevertheless it is considered necessary, there should be sufficient confidence that the available data in terms of PFS, OS, and any other important secondary endpoints will be convincing enough from a scientific and regulatory point of view to meet the objectives of the trial and to ensure that adequate conclusions can be drawn*’ [22]. Fleming et al. argue that ‘*a cross control patients into the experimental therapy at disease progression is only justified when that therapy already has been proven to be an effective rescue treatment*’, which was not the case for tivozanib when setting the protocol [20]. Thirdly, one cannot exclude that sorafenib could be more effective in improving OS, or that tivozanib could have greater cumulative toxicity contributing to the shorter survival [5]. Indeed, Prasad et al. suggested that unequal dose reductions – with a higher dose reduction for sorafenib – may have played a role in PFS results by disadvantaging sorafenib [23]. These reasons could explain the rejection of the approval by the FDA, and they are in line with the comments expressed in the divergent positions that are annexed to the EPAR. In this document, some members of the CHMP listed several of the aforementioned limits and concluded that the ‘*efficacy of tivozanib is not considered robustly demonstrated*’ [8]).

In a context of general demand for replication, the level of evidence provided by at least two pivotal trials is considered as the standard for regulatory approval [24]. EMA’s document entitled ‘*points to consider on application with 1. meta-analyses; 2. one pivotal study*’ describes circumstances in which a single pivotal trial might be sufficient to support approval [25]. This document recalls that ‘*in the exceptional event of a submission with only one pivotal study, this has to be particularly compelling with respect to internal and external validity, clinical relevance, statistical significance, data quality, and internal consistency*’. The document also points out that ‘*all-important endpoints* [should show] *similar findings.*’ So according to the EMA criteria, the TIVO-1 trial is not sound enough to support an approval based on a single pivotal study.

Before the submission of a New Drug Application, the FDA recommended that AVEO conduct a second pivotal clinical trial. This recommendation was in fact concealed by AVEO for many months, resulting in a class action by investors. In the complaint, it is mentioned that AVEO asked whether the second study could be performed as a post-marketing requirement, a demand that was refused by the FDA [26]. This could partly explain why TIVO-3 was slow to start, with the first inclusion taking place 3.5 years after the FDA recommendation. The results of TIVO-3 were finally available in 2019 at a time when it would be considerably difficult to find a role for tivozanib, as too many attractive alternatives, including immune-checkpoint inhibitors, had emerged [27].

In oncology, many cancer drugs are approved based on PFS benefits without evidence of a survival gain [28]. Post-marketing studies are advised to clarify the drug’s effect on OS but such studies do not systematically prove OS benefit or in some instances, do not test or report OS results [29]. So, to limit the risk of a detrimental effect on OS, we deem important that the following issues be taken into consideration: (i) patient-important health outcomes such as quality of life must be encouraged; (ii) surrogate endpoint such as PFS must be used only if a strong association with OS has been demonstrated; (iii) one-way crossover should be avoided so as to not jeopardise overall survival; (iv) through pharmacovigilance analysis, a detrimental effect on mortality should not be suspected; (v) update results of OS must be properly planned, (vi) decision-makers should require post-marketing studies on survival; (vii) absence of evidence on OS must be reported in a transparent manner to patients; (viii) marketing authorization should be reassessed in light of (absence of) post-marketing studies.

With more than 200 citations, the TIVO-1 paper is regarded as a highly cited paper in Web of Science Core Collection. One major endpoint of secondary sources is to transfer knowledge. It is therefore important that secondary sources report clinical study results with accuracy and transparency. As discussed, TIVO-1 has several study limitations that make the results difficult to interpret and inconclusive. It is therefore expected that these limitations are explained in secondary sources to give readers a quick and faithful overview of the level of evidence. Among the 229 citing articles, around one third do not provide any insight into TIVO-1 results and were therefore excluded from the analysis. Among the 151 remaining articles, spin was detected in two out of five articles. By claiming the beneficial effect of tivozanib without taking into account the several limitations, and/or by selectively reporting only statistically significant results, and/or by claiming that the lack of gain in OS was undoubtedly to be attributed to imbalance in post-trial treatments, these secondary sources can mislead the reader, who could over-estimate the efficacy of tivozanib in RCC [15]. In addition, although the following was not considered as spin, twenty-six additional articles suggested that the OS results were ‘likely’ to be confounded by imbalance in post-trial treatments without suggesting further hypotheses. In addition to biased citations, 32 articles cite PFS and OS results in their raw form, *i.e.* without contextualisation or analysis. To a slightly lesser extent, these uncritical citations could also contribute to a misleading impression of a proven efficacy of tivozanib. Interestingly, the FDA’s refusal was far less commented than the EMA’s approval. One limitation of this citation analysis is that assessment of spin is inherently a subjective task. To deal with this issue, the presence of spin was evaluated by two independent reviewers according to standard data extraction methods and discrepancies were resolved through discussion until consensus was reached.

Many shortcomings, such as publication bias or outcome reporting bias, contribute to distorting the evidence from clinical studies in the medical literature [30]. The major consequences are the possible threat to the validity of meta-analyses and reduced reliability of available evidence for decision-making. Several positive initiatives have developed concrete solutions to reduce publication bias and/or outcome reporting bias, but they are mainly designed to improve the quality of primary sources, i.e. original articles on health research studies [31, 32]. While much effort has been invested in improving the quality of original articles [31, 32], there is – to our knowledge – no initiative aiming to reduce citation bias or improve the quality of secondary sources. However, our study, limited to a single clinical trial, shows that incomplete citations or insufficiently critical citations can also affect readers’ interpretations, and lead to overestimating the efficacy of a treatment. This type of citation bias could influence clinicians towards using insufficiently criticised controversial treatments.

## Conclusion

The pivotal TIVO-1 trial is the only one that allowed the EMA to grant tivozanib approval as a first-line treatment in the management of RCC. This trial demonstrates an improvement in PFS for patients treated with tivozanib without improving OS. Although these results suggest a promising treatment, the trial has limitations that do not provide sufficient evidence on the efficacy of tivozanib as a first-line treatment. The broad dissemination of TIVO-1 results in the scientific literature lacked information on the trial characteristics that are essential for all interpretations of results. This literature was widely affected by spin or by uncritically presented results, questioning the reliability of secondary sources.

## Supplementary Information

**Additional file 1: Figure S1.** Co-authorship network of researchers who published articles citing the target article (Motzer et al., 2013 [6]).

**Additional file 2: Table S1.** Electronic search.

**Additional file 3: Table S2.** Main characteristics of citing articles.

**Additional file 4: Table S3.** Quotes from the citing articles illustrating the oversimplification of TIVO-1 results.

## Data Availability

The data that support the findings of this study are available on request from the corresponding author.

## Abbreviations

CHMP: Committee for Medicinal Products for Human Use
EAU: European Association of Urology
EMA: European Medicines Agency
EPAR: European Public Assessment Report
ESMO: European Society for Medical Oncology
FDA: Food and Drug Administration
ORR: Objective Response Rate
OS: Overall Survival
PFS: Progression-Free Survival
RCC: Renal Cell Carcinoma
RECIST: Response Assessment Criteria In Solid Tumors
VEGFR: Vascular Endothelial Growth Factor Receptor
