# Supplementary figures and images for: Tivozanib in renal cell carcinoma: a systematic review of the evidence and its dissemination in the scientific literature
Source: BMC Cancer. 2022 Apr 9;22:381. doi: 10.1186/s12885-022-09475-7 (PMC8994226; doi:10.1186/s12885-022-09475-7)

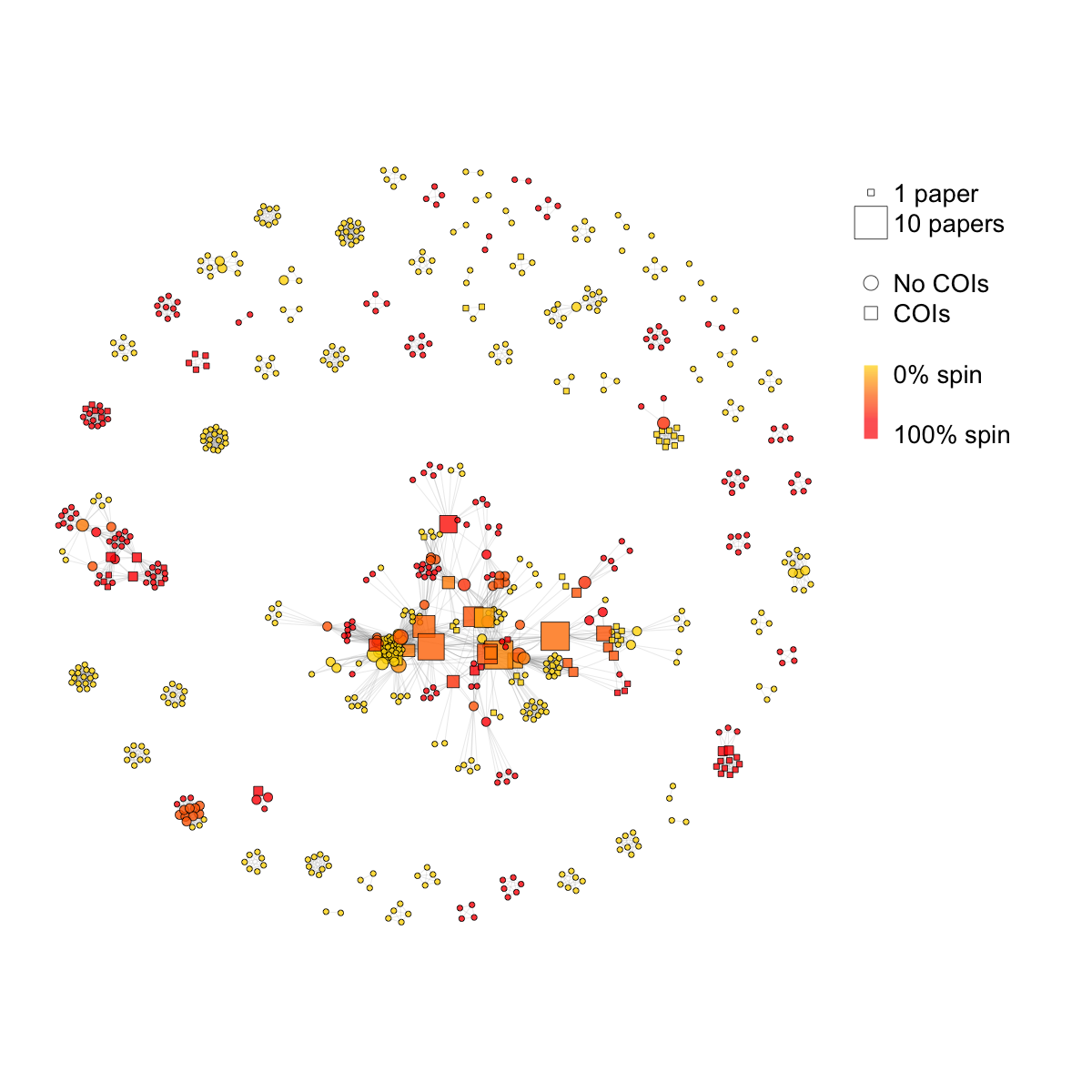

Supplement: Supplementary file 1 — Additional file 1: Figure S1. Co-authorship network of researchers who published articles citing the target article (Motzer et al., 2013 [6]). [file 12885_2022_9475_MOESM1_ESM.tiff]
