# Supplementary material for: Tivozanib in renal cell carcinoma: a systematic review of the evidence and its dissemination in the scientific literature
Source: BMC Cancer. 2022 Apr 9;22:381. doi: 10.1186/s12885-022-09475-7 (PMC8994226; doi:10.1186/s12885-022-09475-7)
Supplement: Supplementary file 2 — Additional file 2: Table S1. Electronic search. [file 12885_2022_9475_MOESM2_ESM.pdf]

Supplementary table 1: Electronic search.

	Equation	Occurrences (December 16, 2020)
**Google Scholar**	(tivozanib OR AV-951 OR KRN-951) AND renal AND random*	564
**Medline**	(tivozanib OR AV-951 OR KRN-951)	124
**Cochrane**	tivozanib	60
**Clinical Trial**	tivozanib	36
