# Supplementary material for: Tivozanib in renal cell carcinoma: a systematic review of the evidence and its dissemination in the scientific literature
Source: BMC Cancer. 2022 Apr 9;22:381. doi: 10.1186/s12885-022-09475-7 (PMC8994226; doi:10.1186/s12885-022-09475-7)
Supplement: Supplementary file 3 — Additional file 3: Table S2. Main characteristics of citing articles. [file 12885_2022_9475_MOESM3_ESM.pdf]

Supplementary Table 2: Main characteristics of *citing articles.*

	N = 151
Publication year	
2013	8 (5.3%)
2014	28 (18.5%)
2015	18 (11.9%)
2016	15 (9.9%)
2017	15 (9.9%)
2018	17 (11.3%)
2019	24 (15.9%)
2020	26 (17.2%)
Publication type	
Experimental articles	35 (23.2%)
Non-experimental articles	116 (76.8%)
Publication language	
English	138 (91.8%)
German	8 (5.5%)
French	2 (1.4%)
Russian	2 (1.4%)
Hungarian	1 (0.7%)
Journal	
Future Oncol	9 (6.0%)
Expert Rev Anticancer Ther	6 (4.0%)
Int J Mol Sci	5 (3.3%)

Expert Opin Pharmacother	5 (3.3%)
J Clin Oncol	5 (3.3%)
Other journals (≤ 4 articles)	121 (80%)
Impact factor 2019	
Median [min – max]	3.562 [0.052 – 33.752]
NA	24
At least one author declares a conflict of interest	
Aveo Pharmaceuticals	20 (13.2%)
Astellas	31 (20.5%)
