# Supplementary material for: Tivozanib in renal cell carcinoma: a systematic review of the evidence and its dissemination in the scientific literature
Source: BMC Cancer. 2022 Apr 9;22:381. doi: 10.1186/s12885-022-09475-7 (PMC8994226; doi:10.1186/s12885-022-09475-7)
Supplement: Supplementary file 4 — Additional file 4: Table S3. Quotes from the citing articles illustrating the oversimplification of TIVO-1 results. [file 12885_2022_9475_MOESM4_ESM.pdf]

Supplementary Table 3: Quotes from the citing articles illustrating the oversimplification of TIVO-1 results.

Citing paper (PMID or DOI)	Paper	Quote	Spin reason	Publication Year
24556857	Non-Experimental	Conversely, the presence of cytokine pretreated patients was largely reported in other two phase III trials that validated the efficacy of pazopanib and tivozanib as up-front targeted agents in mRCC.	Non-disputed efficacy	2014
31178240	Experimental	Anti-vascular endothelial growth factor (VEGF) tyrosine kinase inhibitors (TKIs), such as sunitinib, sorafenib, pazopanib, axitinib, and tivozanib showed high activity and efficacy with a manageable safety profile in the treatment of metastatic RCC (mRCC), and obtained FDA (Food and Drug Administration) and EMA (European Medicines Agency) approvals.	Non-disputed efficacy	2019
10.1007/s12254-019-00545-4	Non-Experimental	Tivozanib was licensed in 2017 by the EMA with a positive phase III trial against sorafenib, already published in 2013.	Non-disputed efficacy	2019
32497970	Non-Experimental	Since then, several targeted therapies have demonstrated improvements for patients with mRCC with positive phase III randomized controlled trial (RCT) evidence for first-line therapy with pazopanib, sorafenib, temsirolimus and tivozanib.	Non-disputed efficacy	2020

31646776	Non-Experimental	In a phase III TIVO-1 study (Table 2), tivozanib compared with sorafenib as first-line targeted therapy in 517 patients with mccRCC. Median PFS was longer with tivozanib than with sorafenib in the overall population (11.9 vs 9.1 mo, HR 0.797, p=0.042). ORR 33.1% vs 23.3% (p=0.014). Median OS was almost similar in the 2 arms (29.3 vs 28.8 mo, HR 1.245, p=0.014). Tivozanib was approved by EMA (Aug 2017) as first-line treatment for mRCC, but not by FDA, due to the lack of significant advantage, in terms of survival, compared to sorafenib.	Non-disputed efficacy & inversion of median OS	2019
26606910	Non-Experimental	The TIVO-1 trial compared tivozanib with sorafenib as first-line therapy. In analyses of all patients (n = 517), tivozanib was superior to sorafenib in terms of median PFS (11.9 vs 9.1 months; HR: 0.797; 95% CI: 0.639–0.993; p = 0.042). Tivozanib showed a consistent advantage to sorafenib in all patient subgroups except those with poor risk in whom sorafenib was associated with longer PFS, although this group comprised only 27 patients.	OS results were omitted	2015
32234426	Experimental	One Phase III study showed that tivozanib was generally well tolerated, and significantly improved progression-free survival (PFS) compared with sorafenib (VEGFR inhibitor) in patients	OS results were omitted	2020

		with metastatic RCC		
24693873	Non-Experimental	Frequent (61%) crossover for patients who experienced progression on sorafenib led to receive tivozanib and accounts for the lack of gain in OS.	OS confounded by crossover	2014
26886011	Non-Experimental	These results were confounded by the fact that patients who had progressed on sorafenib had been allowed to switch to tivozanib but not vice versa.	OS confounded by crossover	2016
30084668	Non-Experimental	Importantly, all OS analyses from the trial were confounded by the one-way crossover design, whereby patients whose disease progressed during sorafenib treatment were permitted to cross over to tivozanib, while patients whose disease progressed during tivozanib treatment received standard-of-care therapies that were available in the respective countries.	OS confounded by crossover	2018
24297950	Experimental	In patients previously untreated with VEGF or mTOR inhibitors, a phase III trial (TIVO-1) demonstrated a significant PFS benefit with tivozanib compared with sorafenib, but no difference in OS.	Uncritical citation	2014
